# Tetanus Toxin Hc Fragment Induces the Formation of Ceramide Platforms and Protects Neuronal Cells against Oxidative Stress

**DOI:** 10.1371/journal.pone.0068055

**Published:** 2013-06-27

**Authors:** Roger Cubí, Ana Candalija, Arturo Ortega, Carles Gil, José Aguilera

**Affiliations:** 1 Departament de Bioquímica i Biologia Molecular and Institut de Neurociències, Universitat Autònoma de Barcelona, Bellaterra, Catalunya, Spain; 2 Departamento de Genética y Biología Molecular, Cinvestav-IPN, México DF, Mexico; 3 Centro de Investigación Biomédica en Red sobre Enfermedades Neurodegenerativas (CIBERNED), Barcelona, Spain; Institute Pasteur, France

## Abstract

Tetanus toxin (TeTx) is the protein, synthesized by the anaerobic bacteria *Clostridium tetani*, which causes tetanus disease. TeTx gains entry into target cells by means of its interaction with lipid rafts, which are membrane domains enriched in sphingomyelin and cholesterol. However, the exact mechanism of host membrane binding remains to be fully established. In the present study we used the recombinant carboxyl terminal fragment from TeTx (Hc-TeTx), the domain responsible for target neuron binding, showing that Hc-TeTx induces a moderate but rapid and sustained increase in the ceramide/sphingomyelin ratio in primary cultures of cerebellar granule neurons and in NGF-differentiated PC12 cells, as well as induces the formation of ceramide platforms in the plasma membrane. The mentioned increase is due to the promotion of neutral sphingomyelinase activity and not to the *de novo* synthesis, since GW4869, a specific neutral sphingomyelinase inhibitor, prevents neutral sphingomyelinase activity increase and formation of ceramide platforms. Moreover, neutral sphingomyelinase inhibition with GW4869 prevents Hc-TeTx-triggered signaling (Akt phosphorylation), as well as the protective effect of Hc-TeTx on PC12 cells subjected to oxidative stress, while siRNA directed against nSM2 prevents protection by Hc-TeTx of NSC-34 cells against oxidative insult. Finally, neutral sphingomyelinase activity seems not to be related with the internalization of Hc-TeTx into PC12 cells. Thus, the presented data shed light on the mechanisms triggered by TeTx after membrane binding, which could be related with the events leading to the neuroprotective action exerted by the Hc-TeTx fragment.

## Introduction

As crucial constituents of the biological membranes, lipids have a main role in the traffic across the plasma membrane and in the starting of intracellular signaling triggered by extracellular modulators. These properties make lipids evolutionally selected targets for pathogens to modulate host cell processes in order to allow their replication and survival [1]. Specialized microdomains in the biological membranes, called *lipid rafts*, have been clearly involved in triggering signaling from the plasmatic membrane [2] as well as in membrane trafficking [3], including entry of pathogens into target cells. Lipid rafts are formed by the specific association between sphingolipids, cholesterol and a specific subset of proteins, which spontaneously separate from glycerophospholipids in the cell membrane [4], and constitute binding platforms for a series of bacterial pathogens, such as *Neisseria Gonorrhoeae, Pseudomonas Aeruginosa* or *Vibrio Cholerae*, or viruses like influenza virus [5], among other functions. Even further, the binding and endocytosis of some protein toxins released by bacteria also rely on lipid rafts, as is the case of anthrax [6] and tetanus [7] toxins. In some cases, pathogens, such as rhinoviruses, influenza virus or HIV [8], [9], promote a membrane-bound sphingomyelinase (SMase) activity [10], increasing the ceramide content in the membrane, thanks to hydrolysis of sphingomyelin (SM), and leading to the formation of ceramide platforms, which are essential for the entry of the pathogen into the host cell [11]. Several enzymes with SMase activity have been described, being the neutral (nSMase) and the acid sphingomyelinases (aSMase) the most studied [12]. Despite extensive studies, the precise cellular function of each of these sphingomyelinases in sphingomyelin turnover and in the regulation of ceramide-mediated responses is not well understood. In mammals, three Mg^2+^-dependent neutral SMases have been identified (nSM1, nSM2 and nSM3). Among the three enzymes, nSMase2 is the most studied and has been involved in some physiological responses including apoptosis, development and inflammation [13]. In addition, recent studies have also supported a role for SMase activity in membrane trafficking, suggesting that nSMase2 may act in the endosomal compartments giving rise to multivesicular endosomes through formation of intravesicular membranes [14].

Tetanus toxin (TeTx) is a high molecular weight (150 kDa) protein produced by the Gram-positive spore-forming bacilli *Clostridium tetani*, and belongs to the clostridial neurotoxin family, together with the botulinum neurotoxins (BoNTs). TeTx consists in two chains, one of 100 kDa (heavy chain or H chain) and one of 50 kDa (light chain or L chain), both linked by a disulphide bond. The carboxyl-terminal half of the heavy chain, referred to as Hc-TeTx or Hc (50 kDa) is responsible for the binding to the plasmatic membrane of target cells and is essential for the ganglioside-binding activity of TeTx [15]. Isolated Hc retains the ability to be transported retroaxonally, similarly as TeTx does [16], gaining entry into its target cells by means of interaction with lipid rafts [7] and subsequent formation of endocytic vesicles in a clathrin-dependent mechanism [17]. The domain organisation of clostridial neurotoxin correlates with a four-step model of intoxication [18]. First, the binding domain (Hc) interacts with the presynaptic nerve terminal membrane [19]. This interaction is thought to occur through both polysialogangliosides (mainly GT1b, GQ1b and GD1b) and protein receptors [20]. Recently, distinct synaptic vesicle proteins have been indicated as protein receptors for different BoNTs, such as the synaptic vesicle protein SV2 for BoNT/A [21] and BoNT/D [22] or synaptotagmins I and II for BoNT/B [23] and BoNT/G [24], suggesting that these toxins exploit synaptic vesicle endocytosis to enter motor neurons. Regarding the TeTx protein receptors, SV2 has been also identified as a mediator of TeTx entry in neurons, depending on synaptic vesicle recycling [25], but other TeTx-binding proteins has also been described, such as Thy-1 [7]. However, other studies indicate that entry of TeTx into spinal cord neurons was independent on synaptic vesicle recycling [26]. Thus, the identification of the protein receptor for TeTx remains still a matter of debate. Then, the light chain is translocated from the lumen of the endosome to the cytoplasm, gaining access to its cytosolic target, as demonstrated for BoNT/A [27]. The last step involves the cleavage of proteins involved in synaptic vesicle membrane fusion, blocking synaptic release. The BoNTs act at the neuromuscular junction (NMJ) level by cleaving one or more of the three synaptic SNARE proteins (synaptobrevin, SNAP25 and Syntaxin-1), depending on the BoNT isotype, while TeTx enters the nervous system via presynaptic terminals of the α-motor neuron at the NMJ, and subsequently undergoing retrograde transport into the spinal cord. There, TeTx enters into the second level inhibitory interneuron and cleaves synaptobrevin [28]. The impairment of inhibitory neurotransmitter release causes spastic paralysis and death of the host. This capability of TeTx and Hc to be transported from the peripheral nervous system, by-passing the brain-blood barrier, has aroused the use of TeTx- or Hc-coupled chimeras as a system to deliver therapeutic agents to the central nervous system [29].

Another action exerted by Hc-TeTx in *in vitro* and *in vivo* models is the inhibition of apoptosis under stress situations or pathological conditions, thanks to the activation of signaling cascades involved in survival [30], [31]. Thus, Hc-TeTx prevents the death of granular neurons in culture due to potassium withdrawal [32] or due to acute treatment with 1-methyl-4-phenylpyridinium (MPP^+^) [33]. Moreover, Hc-TeTx also prevents dopaminergic degeneration and improves motor behavior in rats with unilateral striatal MPP^+^-lesions [34]. Similarly, intramuscularly injected DNA encoding for Hc-TeTx delays the onset of motor symptoms and improves functional deficits, spinal motor neuron survival and lifespan in an animal model of amyotrophic lateral sclerosis (ALS), the SOD1G93A transgenic mice strain [35].

Thus, we explored the hypothesis that Hc, as responsible for TeTx membrane binding and endocytosis, could increase the ceramide content in the membrane of host cells by means of SMase activity enhancement. In the present report we demonstrate that the incubation of cultured granule neurons or of PC12 cells with Hc increases the ceramide/sphingomyelin ratio in the target cells. In addition, Hc enhances the SMase activity, which is reverted by pretreatment with GW4869, a specific inhibitor for nSMase. The Hc-activated nSMase activity leads to the formation of ceramide platforms in the plasma membrane, but is not essential for the internalization of Hc into target cells. On the contrary, nSMase activity is necessary for the Hc-triggered signaling and, more interestingly, for the promotion of target cell survival under oxidative stress, a new capacity never described before for Hc.

## Materials and Methods

### Cellular Cultures and nSM2 Knockdown

Cerebellar granule neurons (CGN) cultures and cultured cortical neurons (CCN) were prepared as described in [33] and in [32], respectively. PC12 cells were obtained from ATCC (CRL-1721) and cultured in DMEM supplemented with 10% horse serum, 5% fetal calf serum, 50 U/mL penicillin and 50 *µ*g/mL streptomycin, at 37°C in a humidified 5% CO_2_ atmosphere. For experiments, cells were seeded at a density of 3,5·10^6^ cells/cm^2^ in plates coated with 10 *µ*g/mL poly-L-lysine. After 24 h, the medium was supplemented, when indicated, with NGF 50 ng/mL (Sigma). Supplemented medium was changed every 3–4 days, and cells were used after 7 days of NGF treatment. Recombinant Hc-TeTx fragment was obtained as previously reported [31]. Murine NSC-34 cells were cultured in DMEM with 10% fetal bovine serum and 1% penicillin and streptomycin. For knockdown assays, cells were cultured for 24 h, and siRNA was transfected using Lipofectamine 2000 (Invitrogen) according to the manufacturer’s instructions. Cells were transfected with functional, non-targeting control siRNA (All Stars Negative Control from Qiagen) or a mix of four pre-designed specific Flexitube siRNAs against the *smpd3* gen (Qiagen). For each well, 5 pmol of each siRNA was added. Cells were then incubated for an additional 72 h prior to the experiments. Decrease of the neutral sphingomyelinase-2 (nSM2) protein content was assessed by means of western blot. Labeling of Hc-TeTx with Alexa Fluor 488 maleimide was performed according to the kit manufacturer’s instructions (Invitrogen), obtaining an average of 1.8 moles of dye per mole of Hc-TeTx.

### Measurement of Cell Viability

PC12 cells were plated at a density of 1 10^5^ cells/mL in 24-well plates, while NSC-34 cells were plated at 2.5 10^3^ cells/mL in 24-well plates. Cell viability was determined by using the conventional 3-(4,5-Dimethylthiazol-2-yl)-2,5-diphenyltetrazolium bromide (MTT) reduction assay. In the MTT assay, the viable cells convert cell-permeable soluble dye MTT to insoluble blue formazan crystals and this reaction is catalyzed by the succinate dehydrogenase, a mitochondrial respiratory chain enzyme easily inactivated by oxidative stress. After incubation with the indicated compounds, cells were treated with MTT solution (1 mg/mL final concentration) for 2 h at 37°C. The dark blue formazan crystals formed inside the intact mitochondria were solubilized with dimethylsulfoxide, and the absorbance measured at 570 nm using a microplate reader (TECAN GmbH, Salzburg, Austria).

### Sphingolipid Determination by ^14^C-labeling and Thin Layer Chromatography

In the case of CGN, cells were treated after 7 DIV, while CCN were treated after 11 DIV. In the case of NGF-differentiated PC12, 1·10^5^ cells/mL were treated after NGF treatment (50 ng/mL) for 6 days. In both cases cells were incubated overnight with ^14^C-serine (1 µCi/mL) previously to the treatment, in order to label the sphingolipids. After every treatment the medium was aspired and two washes with PBS were performed. Lipids were immediately extracted with incubation in 1.2 mL of a mixture consisting in chloroform and methanol (1∶2) for 15 min at -20°C. Subsequently, 0.5 mL of chloroform and 0.5 mL of water were added. After shaking the mixture, tubes were centrifuged (1,000 rpm, 5 min) and the organic phase was extracted. As internal standard, 20 µg of ceramide were added in every sample, then evaporated and resuspended in 25 µl of chloroform/methanol (4∶1). The lipid extracts were then resolved on silica high-performance TLC plates using chloroform/methanol/water (68∶28:4). Ceramide and sphingomyelin standards were resolved in parallel and staining of lipids was performed using iodine. Bands from samples corresponding to ceramide and sphingomyelin were scrapped and ^14^C radioactivity measured in a scintillation counter.

### Sphingomyelinase Activity Analysis

After treatment, cells were harvested with 500 µl of 1× reaction buffer (Tris-HCl 0.1 M, pH 7.4, 10 mM MgCl_2_, 1% Triton X-100 and protease/phosphatase inhibitors), sonicated and centrifuged at 9,000 g for 30 min at 4°C. The supernatant fraction was used for neutral Sphingomyelinase (nSMase) activity by using the Amplex Red Sphingomyelinase Assay Kit, according to the manufacturer’s instructions (Molecular Probes, UK). The fluorescence was determined with a Series BioAssay Reader (PerkinElmer Instrument).

### Immunocytochemistry

The cells were cultured on coverslips, previously treated with polylysine (Sigma), were fixed with 4% paraformaldehyde for 20 min and washed three times with phosphate-buffered saline (PBS). The fixed cells were first incubated for 1 h at room temperature with blocking solution (PBS, Triton X-100 0.01%, gelatine 0.2% and fetal bovine serum 10%) and then with primary antibody in the same buffer (but only with 1% of FBS) overnight at 4°C. The anti-ceramide antibody (clone 15B4, from Sigma) was used at 1∶50 dilution. The monoclonal antibody against neutral sphingomyelinase-2 (G6) was purchased by Santa Cruz Biotechnology (CA; USA). Secondary antibodies were Alexa-546-conjugated (Invitrogen), used at a 1∶400 dilution for 1 h at room temperature in the same buffer as the primary antibody. Preparations were washed three times with PBS at room temperature for 5 min and then the coverslips were mounted with fluorescence mounting medium, containing DAPI in some cases (Dako, Denmark). Slides were observed using a Leica TCS SP2 confocal microscope. Fluorescence intensity was measured using the Advanced Fluorescence Lite software from Leica. Tridimensional reconstructions were obtained by acquisition of 18 z-planes by confocal microscopy and subsequent use of the Imaris 6.3.1 software. For internalization experiments, cells were grown in 35-mm glass-bottom microwell dishes (from Ibidi), incubated with 10 nM H_C_-TeTx-Alexa488 and the process followed by time-lapse confocal microscopy for 1.5 h inside a thermostatic chamber at 37°C, with CO_2_ supply. Images of 18 z-planes were acquired every 10 min.

### Detergent-resistant Membranes Extraction

At the end of the incubation, the medium was removed and cells were rinsed with cold PBS and cultures were homogenized at 37°C with 250 uL of lysis buffer containing 1% Brij 98 (Sigma) by end-over-end mixing (30 min, 37°C). Thereafter, the extracts were adjusted to 45% sucrose, and overlaid with 7 mL of 35% sucrose in sodium buffer and 2 mL of 5% sucrose in sodium buffer, inside an ultracentrifugation tube. DRM fractions were isolated by ultracentrifugation at 35,000 rpm, for 18 h, 4°C, using a SW41 rotor (Beckman Instruments Inc.). The gradient was harvested in 10 fractions of 1 mL each.

### Western-blot Analysis

At the end of the incubation, the medium was removed and cells were rinsed with cold PBS. The cells were lysed by scraping them in ice-cold lysis buffer, 62.5 mM Tris (pH 6.8), 2% SDS, 10% (v/v) glycerol, 50 mM dithithreitol, 0.1% Bromophenol Blue, and subjected to SDS/PAGE. The separated proteins were transferred to Protran nitrocellulose membrane (Schleicher and Schuell, Dassel, Germany), using a Mini TransBlot Cell 3 (Bio-Rad, Hercules, CA, U.S.A.) at 100 V for 1 h. The blotting buffer contained 25 mM Tris, 200 mM glycine and 10% (v/v) methanol. The membrane filters were blocked for 1 h with Tris-buffered saline, supplemented with 0.1% Tween 20 and 5% (w/v) defatted powdered milk. Then the membranes were incubated overnight with the corresponding antibody diluted in blocking buffer. Antibodies against phosphoSer473-Akt and against total Akt were purchased by Cell Signaling Tech. Next, the membrane filters were incubated for 1 h with a secondary antibody conjugated with horseradish peroxidase diluted in blocking buffer. Several washes with Tris-buffered saline/0.1% Tween 20 were performed between all of the steps. The Western blots were developed using ECLR detection reagents from Amersham Pharmacia Biotech (Little Chalfont, Bucks., U.K.) and exposed to Amersham ECLR hyperfilms.

## Results

### Hc Fragment Induces a Moderate but Rapid and Sustained Increase in the Content and in the Ceramide/Sphingomyelin Ratio due to Promotion of Neutral Sphingomyelinase Activity

**Figure 1:**
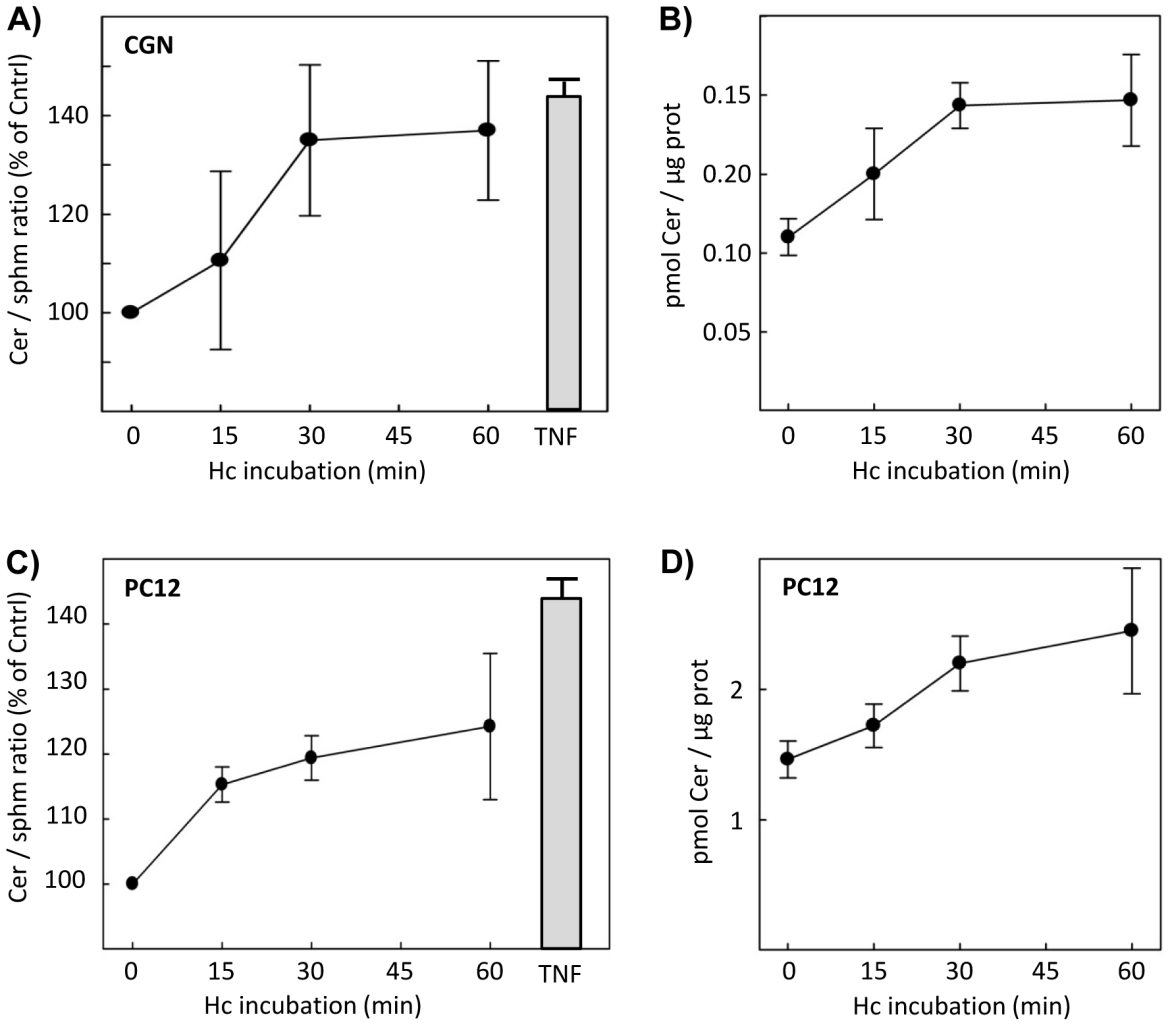
Hc-TeTx induces a moderate but rapid and sustained increase in the ceramide content and in the ceramide/sphingomyelin ratio. Sphingolipids in cultured granular neurons (CGN) or in NGF-differentiated PC12 were labeled by incubation of the cells with ^14^C-serine for 24 h. Subsequently, cultureswere treated with Hc-TeTx 10 nM at the indicated time points, or with TNF (50 ng/mL) for 6 h as a positive control. Radioactivities incorporated in ceramide and sphingomyelin were measured after TLC separation, as indicated in Materials and Methods. Amounts of ceramide after every treatment were determined and expressed in terms of pmols of ceramide/µg of protein **(A and C)**. The ceramide/sphingomyelin ratios were also measured **(B and D)**. Shown are mean ± SD of three independent experiments.

**Figure 2:**
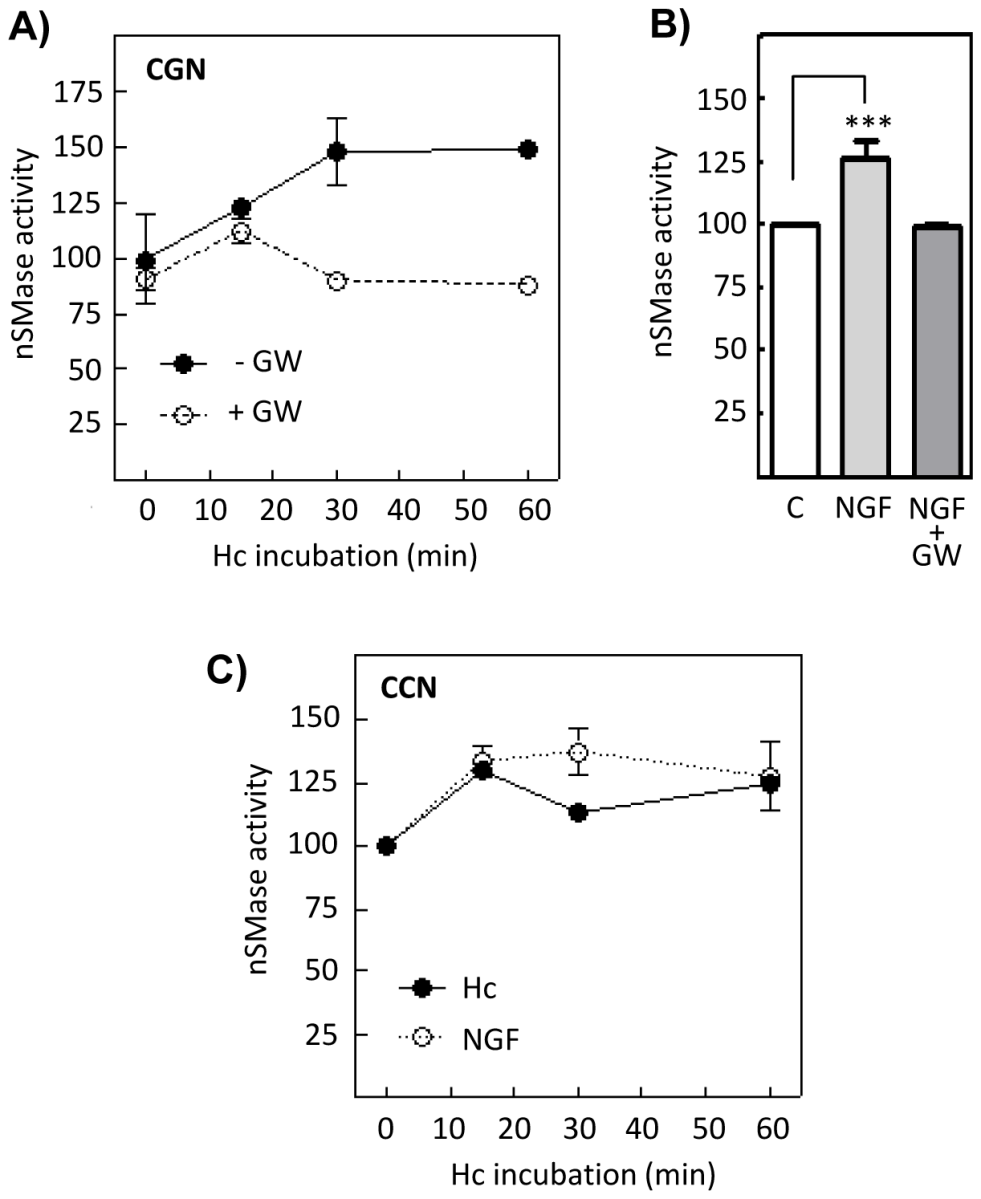
Hc-TeTx induces nSMase activity. **A)** CGN were incubated at different times with 10 nM Hc-TeTx and SMase activity was measured at pH 7.5 using the Amplex Red Sphingomyelinase Assay Kit (Molecular Probes, UK). Alternatively, cells were pretreated for 1 h with 20 µM GW4869, a specific inhibitor for nSMase, previously to the Hc treatment. Each point represents the mean ± SD of three independent experiments. **B)** The graphic shows the increase in nSMase activity after 1 h of incubation with NGF 50 ng/mL, which is totally prevented by pretreatment with 20 µM GW4869. Shown are mean ± SD of three independent experiments. Values were analyzed by one-way ANOVA followed by Bonferroni post hoc test to compare SMase activity in Hc-treated cells respect to controls. ***P<0.0001. **C)** Cultured cortical neurons (CCN) were incubated at different times with 10 nM Hc-TeTx or with NGF 50 ng/mL and SMase activity was measured at pH 7.5 using the Amplex Red Sphingomyelinase Assay Kit (Molecular Probes, UK). Each point represents the mean ± SD of three independent experiments.

In order to assess the effect of the Hc fragment on the ceramide levels, sphingolipids in CGN were metabolically labeled by overnight incubation of cultures with ^14^C-serine. Subsequent analyses by TLC show that treatment with 10 nM Hc-TeTx at different times causes a moderate but rapid and sustained increase in the ceramide content detectable at 15 min and maintained for 1 h (figure 1A). Assessment in CGN of the ceramide/sphingomyelin ratio, also using the sphingolipid signals obtained from TLC, shows increases also detectable at 15 min and maintained for 1 h (figure 1B). These results are also observed in NGF-differentiated PC12 cells (figure 1C and 1D). The extents of these increases were similar to those exerted by tumor necrosis factor (TNF), a known enhancer of the ceramide content by means of sphingomyelinase activity promotion [36]. The increase in the ceramide/sphingomyelin ratio indicates appearance of ceramide caused by the promotion of a sphingomyelinase activity, similarly to the TNF case, rather than *de novo* ceramide synthesis. To determine whether the Hc fragment is able to induce SMase activity, CGN were incubated at different times with Hc 10 nM. The results show an increase in the nSMase activity that runs in parallel with the aforementioned increase in the ceramide content shown in figure 1, since nSMase activity increases gradually reaching the maximum at 30 min (figure 2A). Pretreatment with a specific nSMase inhibitor, GW4869, abolishes the Hc-dependent activation (figure 2A), thus corroborating the nSMase-driven hydrolysis. Since NGF has also been described as an enhancer of the nSMase activity in CGN in a short time of incubation [37], this neurotrophin was used as an additional control of the technique, showing an extent of activation similar to that observed with Hc, and being also totally prevented by GW4869 pretreatment (figure 2B). The same experiments were conducted in parallel with Hc and with NGF in cultured cortical neurons (CCN), showing similar results (figure 2C).

### Hc-TeTx Induces the Increase of Ceramide Platform Content in CGN, due to nSMase Action

**Figure 3:**
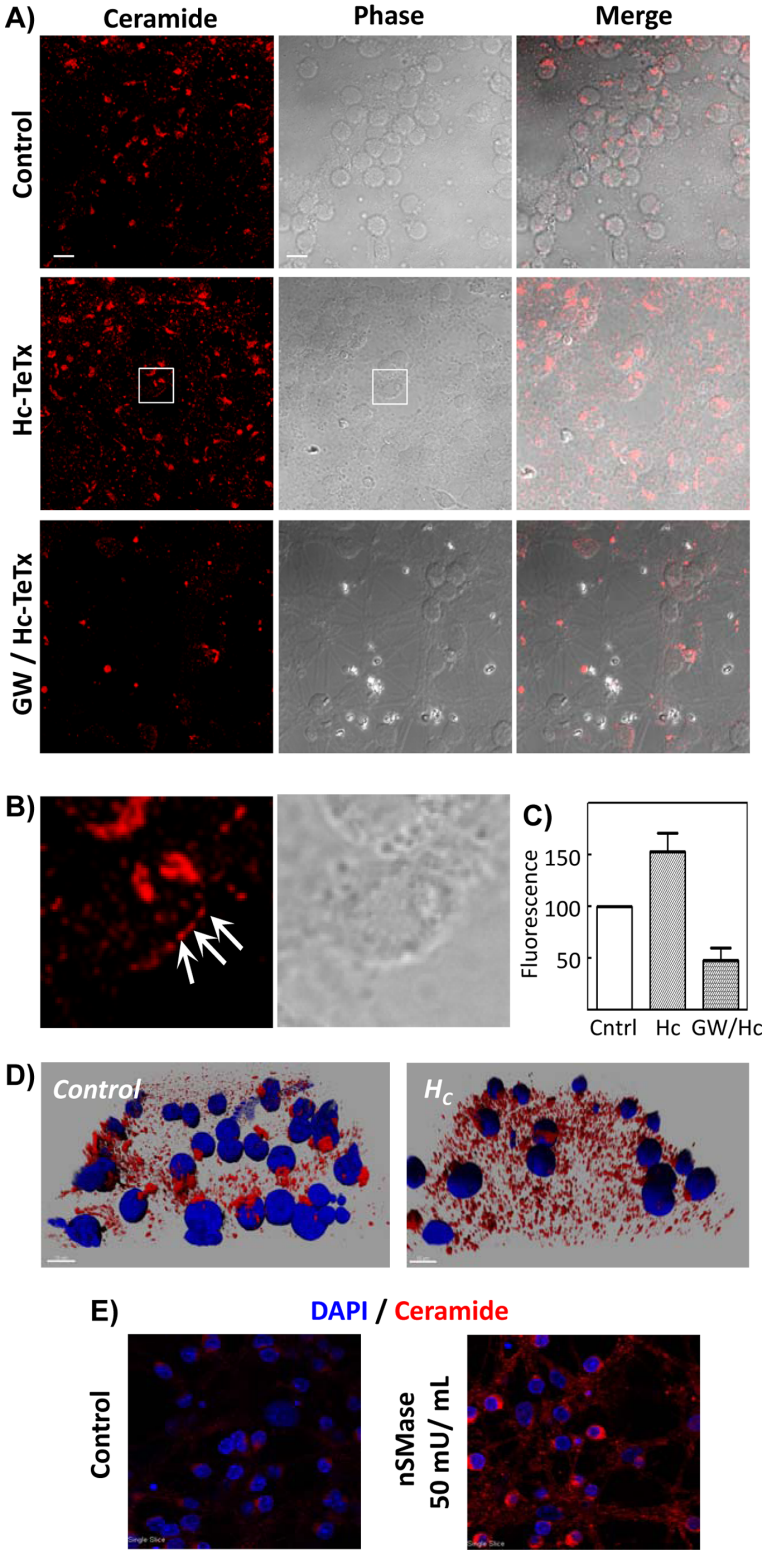
Hc-TeTx induces the increase of ceramide-platform content in CGN, due to nSMase action. **A)** Cells were treated with Hc-TeTx 10 nM for 30 min, with or without pretreatment with 20 µM GW4869 for 1 h. Control cells were left totally untreated. After treatment cells were fixed, stained with anti-ceramide-antibody (clone 15B4) and analyzed by confocal microscopy. As can be seen in A, the results indicate the formation of ceramide-enriched membrane platforms upon Hc treatment, which is prevented by nSMase inhibition with GW4869. Bright field images and merges are also shown. The images are representative for each three independent experiments. Bars in the control images represent 100 µm. **B)** Magnifications of the areas indicated in the Hc-treated cultures shown in section A. Ceramide clusters in the plasma membrane are indicated by arrows. **C)** Quantitative analysis of the formation of ceramide-enriched membrane platforms upon Hc treatment, which indicates nSMase-dependent increase in ceramide-platforms. The assessment of the fluorescence was performed using the Advanced Fluorescence Lite software from Leica. The fluorescence from 18 z planes was measured to obtain every determination. For every condition, 10 fields were measured with a mean of 25 cells per field. The results represent the mean ± SD of three independent studies. **D)** Tridimensional composition of cells without or with treatment with Hc-TeTx 10 nM for 30 min. The nuclei were stained with DAPI (blue) and ceramide was detected with the 15B4 antibody and a secondary antibody coupled to Alexa 546 (red). Fluorescence from 18 z planes was captured by confocal microscopy and tridimensional images were created with Imaris 6.3.1 software. **E)** CGN were treated or not with 50 mU/mL of nSMase from *Bacillus Cereus* for 30 min and subsequently labeled with the anti-ceramide antibody (red) and with DAPI (nuclei).

In order to corroborate the induction of ceramide levels due to the Hc fragment, Hc-treated or untreated CGN were labeled with an antibody specific for ceramide (clone 15B4), as stated in the Materials and Methods section. As seen in figure 3A, untreated cells show a ceramide labeling mainly localized in the cell bodies, probably corresponding to the Golgi apparatus, but very slightly in the neurites. Treatment of cells with 10 nM Hc for 1 h induces a clear enhancement of ceramide labeling. Patches corresponding to ceramide accumulations in the plasma membrane can be observed in the magnification shown in figure 3B. The appearance in a spotted fashion in the cell membrane is in accordance with the described self-aggregation of ceramide molecules to form ceramide-enriched platforms. The signal corresponding to ceramide was determined as stated in the Materials and methods section, showing an increase of approximately 150% of total fluorescence respect to control (figure 3C). Pretreatment with GW4869 totally abolishes the signal due to Hc incubation, demonstrating the nSMase-dependent ceramide platform formation. Tridimensional reconstruction with Imaris software using confocal images corroborates the increase in the ceramide content after Hc treatment, as well as the appearance of a patch-like pattern, clearly visible in the neurite network (figure 3D). The 15B4 antibody specificity is corroborated after incubation of CGN with 50 mU/mL of nSMase from *Bacillus Cereus* for 30 min and subsequently labeling with the 15B4 antibody and with DAPI (Figure 3E), showing a punctuated pattern similar to these seen after Hc treatment.

### Hc-TeTx and nSM2 Show Partial Colocalization in the Plasma Membrane

**Figure 4:**
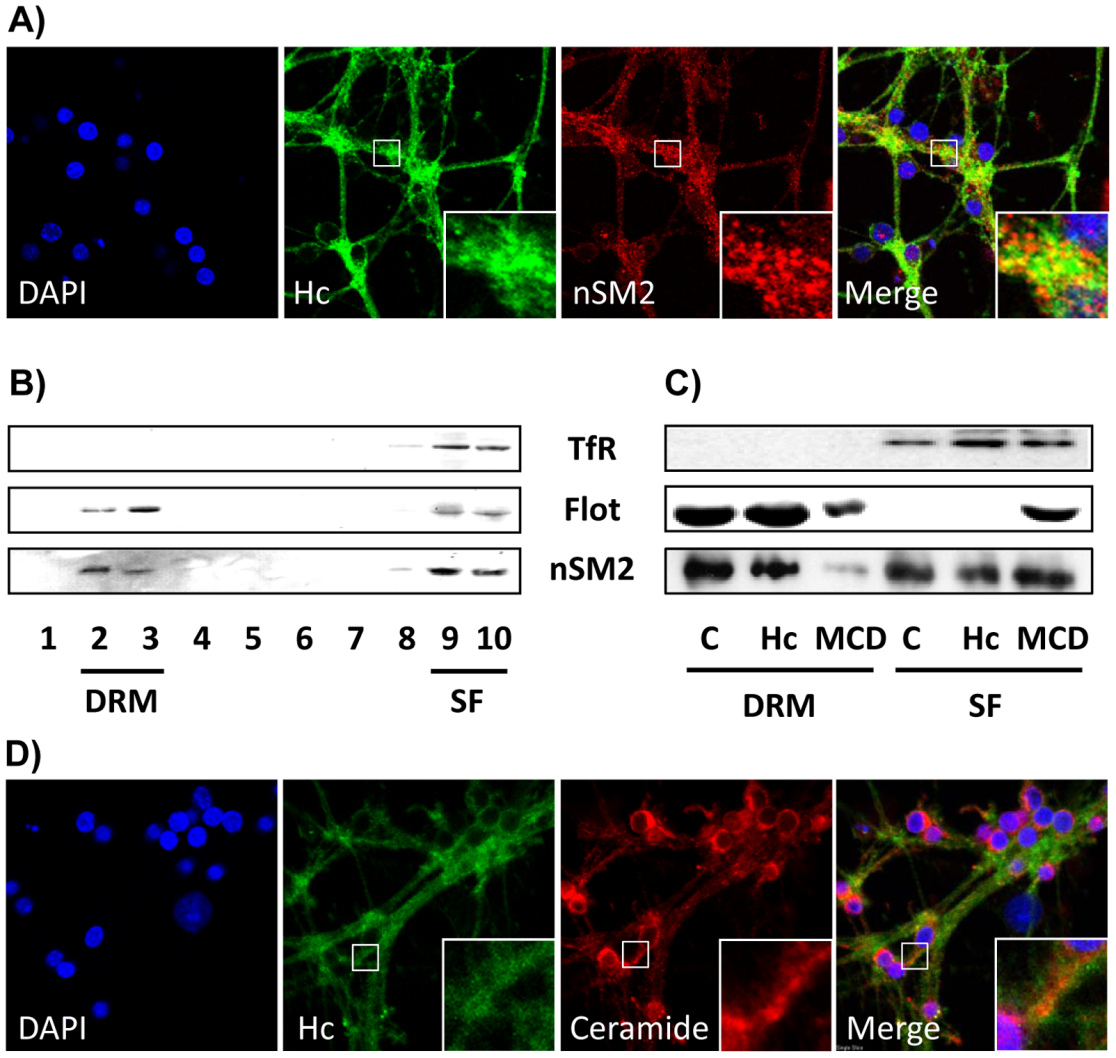
Hc-TeTx and nSM2 show a similar distribution in the plasma membrane. **A)** CGN cultures were incubated with Hc-Alexa 488 (10 nM) for 15 min and subsequently fixed and labeled with the nSM2 antibody. Merge image show that both proteins have a punctuated pattern and partial colocalization, as is highlighted by the magnification. Nuclei are stained with DAPI (blue). **B)** Extraction of detergent-resistant membranes (DRM) from CGN with 1% Brij 98 shows that nSM2 partially associates with membrane rafts, as has been described for TeTx and for Hc. Transferrin receptor (TfR) is used as marker of soluble membrane while Flotillin-1 is used as marker of DRM. **C)** CGN were treated with MCD 5 mM for 30 min, treatment that extracts cholesterol from the membranes and turns DRM sensitive the detergent treatment, as can be seen thanks to the signal increase of both Flotillin-1 and nSM2 in the soluble fractions (SF) respect to the control condition (*C*). Hc treatment (10 nM for 30 min) does not cause any change in nSM2 association to DRM. **D)** CGN cultures were incubated with Hc-Alexa 488 as in A and subsequently fixed and labeled with the anti-ceramide antibody. Merge image show that both proteins have a punctuated pattern and a partial colocalization, as is highlighted in the magnification. Nuclei are stained with DAPI (blue).

CGN cultures were incubated for 60 min with 1 nM Hc-TeTx-Alexa 488 at 37°C, and nSM2 or ceramide subsequently labeled with a sandwich composed of the corresponding primary antibody and the secondary antibody labeled with Alexa 546. After staining of the nuclei with DAPI, confocal images were taken, in order to assess the distribution of each component. The punctuated pattern shown by Hc-TeTx agrees with its described interaction with membrane rafts [7] and it is similar to that shown by nSM2, showing both proteins a partial extent of colocalization, as can be observed in the merge image and in the magnification (figure 4A). Moreover, nSM2 is also observed in the nuclei, as has been already described in the literature [38]. The colocalization between Hc and nSM2 is supported by the observation that nSM2 can be found in detergent-resistant membranes (DRM), extracted from CGN with Brij98, which are, as aforementioned, preferential sites of Hc interaction with the target membrane (figure 4B). In order to assess whether Hc affects the nSM2 localization in rafts, DRM were extracted after Hc treatment for 30 min or after methyl-β-cyclodextrin (MCD) treatment for 30 min. DRM are assumed to be the experimental equivalent of the rafts microdomains, while MCD extracts cholesterol from membranes and turn rafts detergent-sensitive. As can be seen in the figure 4C, MCD (5 mM for 30 min) effectively induces solubilization of flotillin and of nSM2, thus both increasing their content in the soluble fractions (SF) respect to their content in the DRM, result that demonstrates the cholesterol-dependence of the DRM extracted with Brij98 from CGN, while Hc fragment (10 nM for 30 min) does not change the distribution of nSM2 in DRM respect to nSM2 in SF (figure 4C), indicating that promotion of nSMase activity by Hc is not linked to its association to ratfs. In order to assess whether Hc binds specifically to ceramide-enriched domains in the membrane, localizations of Hc and ceramide were determined by confocal microscopy. After 30 min of Hc-Alexa 488 incubation, CGN were also labeled with anti-ceramide and with DAPI. Confocal images show partial colocalization of Hc and ceramide (figure 4D), although is not possible to assess, in the used conditions, whether the detected ceramide was previous to the Hc treatment or a consequence of the nSMase activation. Thus, although Hc can, at some extent, interact with ceramide platforms, these do not seem to be the only sites for membrane binding of Hc.

### nSMase Activity is Essential for Hc-dependent Signaling and for Hc-promoted Survival under Oxidative Stress

**Figure 5:**
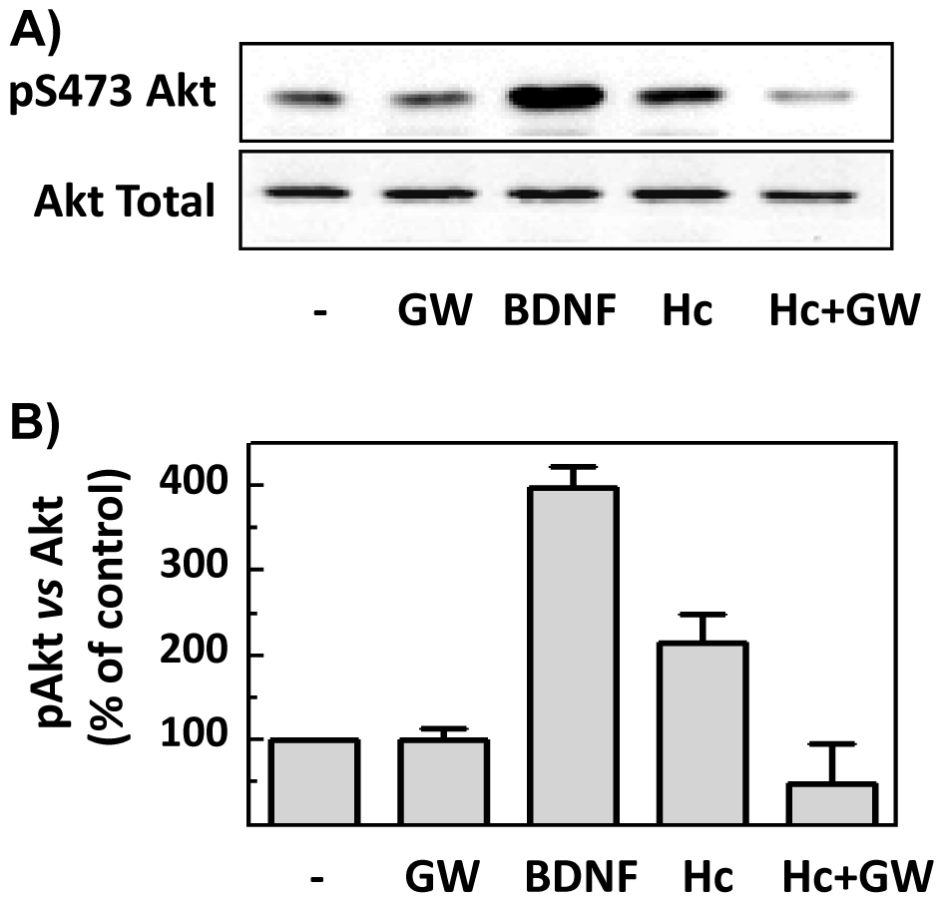
Hc-TeTx-dependent Akt signaling depends on nSMase. **A)** Phosphorylation of Akt in serine 473 in CGN were tested after 10 nM Hc-TeTx treatment for 15 min in low potassium conditions (5 mM), with or without GW4869 (20 µM) pretreatment for 1 h, using 50 ng/mL BDNF for 15 min as positive control and an phosphospecific antibody in western blot. Loading control was assessed using an antibody against total Akt. Experiment was performed in triplicate. **B)** Films were scanned and signal quantified using Image J software. The values are represented in the bar graph in percentage respect to the control condition. Error bars represent SD.

**Figure 6:**
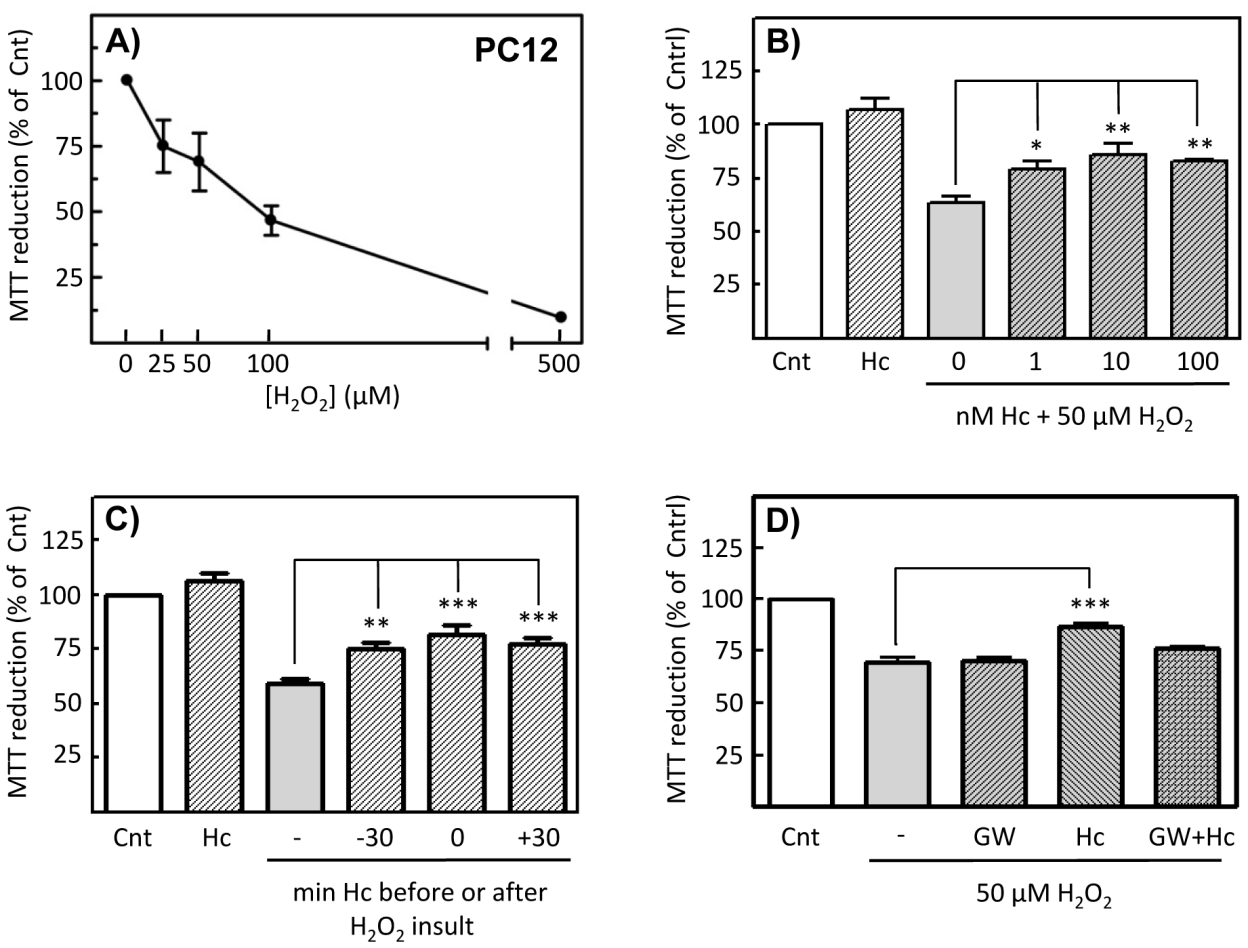
Hc-TeTx protects PC12 cells from oxidative stress, in a nSMase-dependent manner. **A)** PC12 cells were treated with increasing concentration of hydrogen peroxide for 48 hours, in order to determine the optimal conditions to assess the protection performed by Hc-TeTx. Cell viability was assessed by means of the MTT reduction assay. Shown are the mean ± SD. **B)** **C)** PC12 were treated with 50 µM H_2_O_2_, without or with cotreatment with 10 nM Hc-TeTx at different times of application, i.e., 30 min before (-30), at the same time (0) or 30 min after H_2_O_2_ insult (+30), showing an enhancement of cell viability caused by Hc-TeTx in any case. **D)** nSMase involvement in Hc-TeTx-dependent protection was assessed by pretreatment with 20 µM GW4869 previously to H_2_O_2_ insult. In all cases, the values were analyzed by one-way ANOVA followed by Bonferroni post hoc test to compare SMase activity in Hc-treated cells respect to control. *p<0.05, **p<0.01, ***p<0.001.

In previous studies from our group, the promotion of neuronal survival by Hc-TeTx was demonstrated, as has been stated in the Introduction section. Specifically, Hc protects granule neurons against potassium withdrawal [32] or against MPP^+^ insult [33]. Additionally, it was demonstrated that this pro-survival effect is dependent on Akt and ERK-1/2 signaling [32]. In order to determine the role of nSMase in Hc signaling, CGN cultures were treated with or without GW4869 (20 µM for 1 h), before Hc treatment, using BDNF (50 ng/mL for 15 min) as positive control. Results show that the enhancement of Akt phosphorylation in serine 473 after Hc treatment is abrogated by GW action (figure 5A and 5B). Moreover, in order to study the role of Hc-enhanced nSMase activity in cell survival, hydrogen peroxide was used as death-inducer, since oxidative stress due to H_2_O_2_ has been shown in several works to be a cause of apoptosis in PC12 cultures (e.g., [39]). Thus, NGF differentiated-PC12 cells were treated with 50 µM H_2_O_2_, which causes a cell loss of 40% approximately, as assessed by MTT reduction (figures 6A and 6B). Co-treatment of cells with increasing concentrations of Hc together with 50 µM H_2_O_2_, shows that Hc enhances cell viability under oxidative stress (figure 6B). Subsequent experiments in which Hc fragment were applied 30 min before, at the same time or 30 min after oxidative insult show that Hc performs its best protective action when applied together with H_2_O_2_, even though Hc is also significantly effective 30 min after insult (figure 6C). The inhibition of nSMase with GW4869 totally prevents the protective action of Hc applied together with H_2_O_2_, demonstrating an essential role of nSMase activity in Hc-driven protection (figure 6D).

### nSM2 siRNA Knockdown

**Figure 7:**
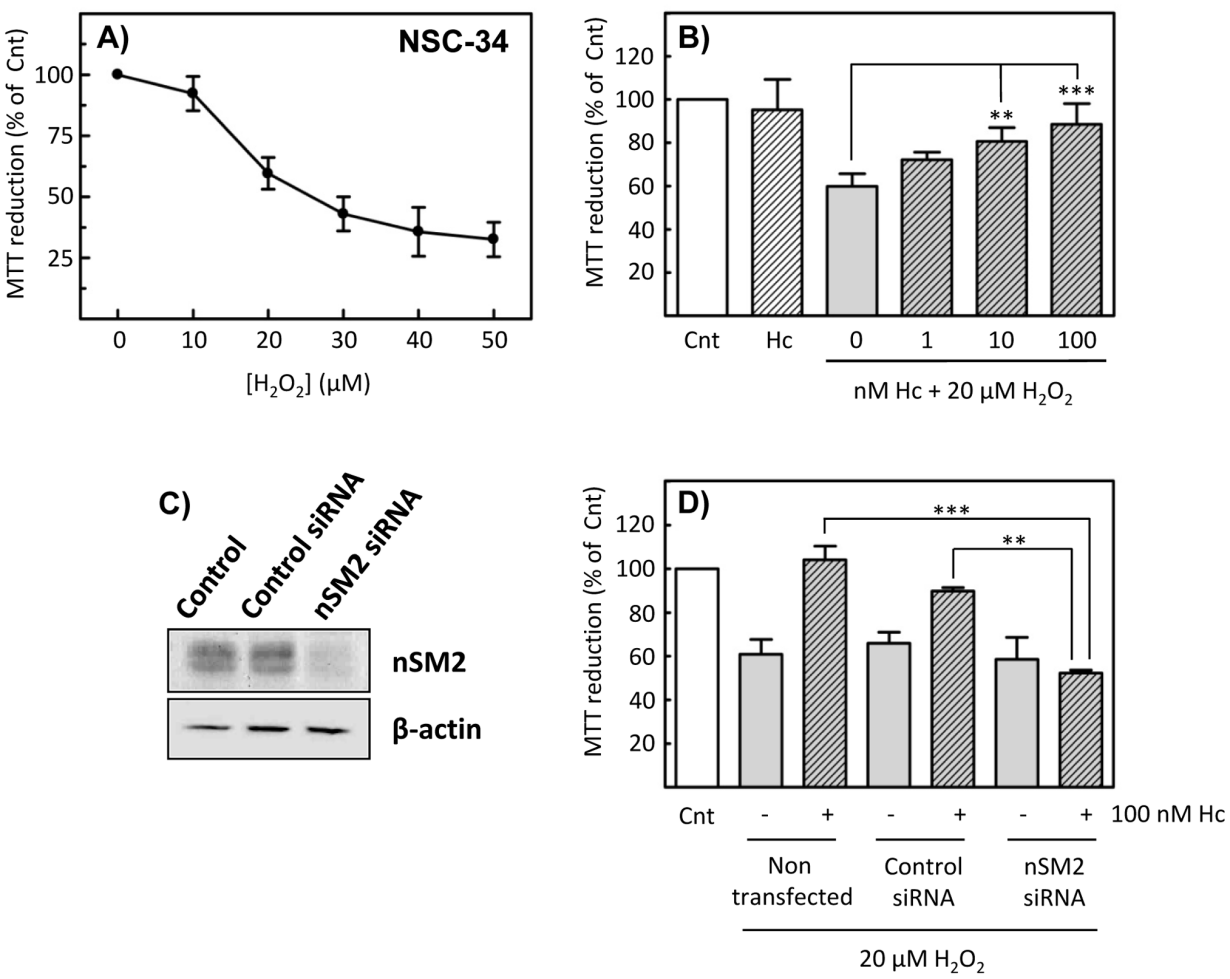
The effect of siRNA knockdown of nSM2 expression on Hc-TeTx-induced viability of NSC-34 cells under oxidative stress. **A)** NSC-34 cells were treated with increasing concentration of hydrogen peroxide for 48 hours, in order to determine the optimal conditions to assess the protection performed by Hc-TeTx. Cell viability was assessed by means of the MTT reduction assay. Shown are the mean ± SD. **B)** NSC-34 cells were treated with 20 µM H_2_O_2_ for 48 hours, without or with cotreatment with increasing concentrations of Hc-TeTx, showing an enhancement of cell viability caused by Hc-TeTx. (Cnt, control). ). Each histogram is the average ± SD from three independent experiments. **p>0.01, ***p<0.001, using one-way ANOVA, followed by the Bonferroni’s post-hoc test. **C)** Short interfering RNAs were used to reduce cellular nSM2 levels (nSM2 siRNA) with a non-silencing siRNA used as control (Control siRNA). Western blots were used to assess the reduction of nSM2 levels. **D)** Treatment of NSC-34 cells with siRNA against nSM2 markedly decreased Hc-TeTx-induced viability of the cells under oxidative stress (20 µM H_2_O_2_), as determined by MTT assay (Cnt, control). Each histogram is the average ± SD from three independent experiments. **p<0.01, ***p<0.001, using one-way ANOVA, followed by the Bonferroni’s post-hoc test.

In order to corroborate the nSMase-dependent cell protection exerted by Hc-TeTx, knockdown of the *smpd3* gene product, i.e. the neutral sphingomyelinase-2 (nSM2) protein, was conducted by means of siRNA. The mouse motorneuron-like hybrid cell line NSC-34 was used in these experiments. A concentration of 20 µM H_2_O_2_ causes a cell loss of 40% approximately, as assessed by MTT reduction (figures 7A). Co-treatment of cells with increasing concentrations of Hc together with 20 µM H_2_O_2_, shows that Hc enhances cell viability under oxidative stress (figure 7B). On the other hand, treatment of NSC-34 cells with siRNA against nSM2 causes massive depletion of the protein (figure 7C). Subsequently, experiments assessing cell viability under oxidative stress were performed, with or without siRNA treatment. Results show that knockdown of nSM2 totally prevents the protective action of Hc applied together with H_2_O_2_, confirming the important role of nSMase activity in Hc-driven protection (figure 7D).

### Neutral Sphingomyelinase Activity is not Crucial in Hc-TeTx Internalization

**Figure 8:**
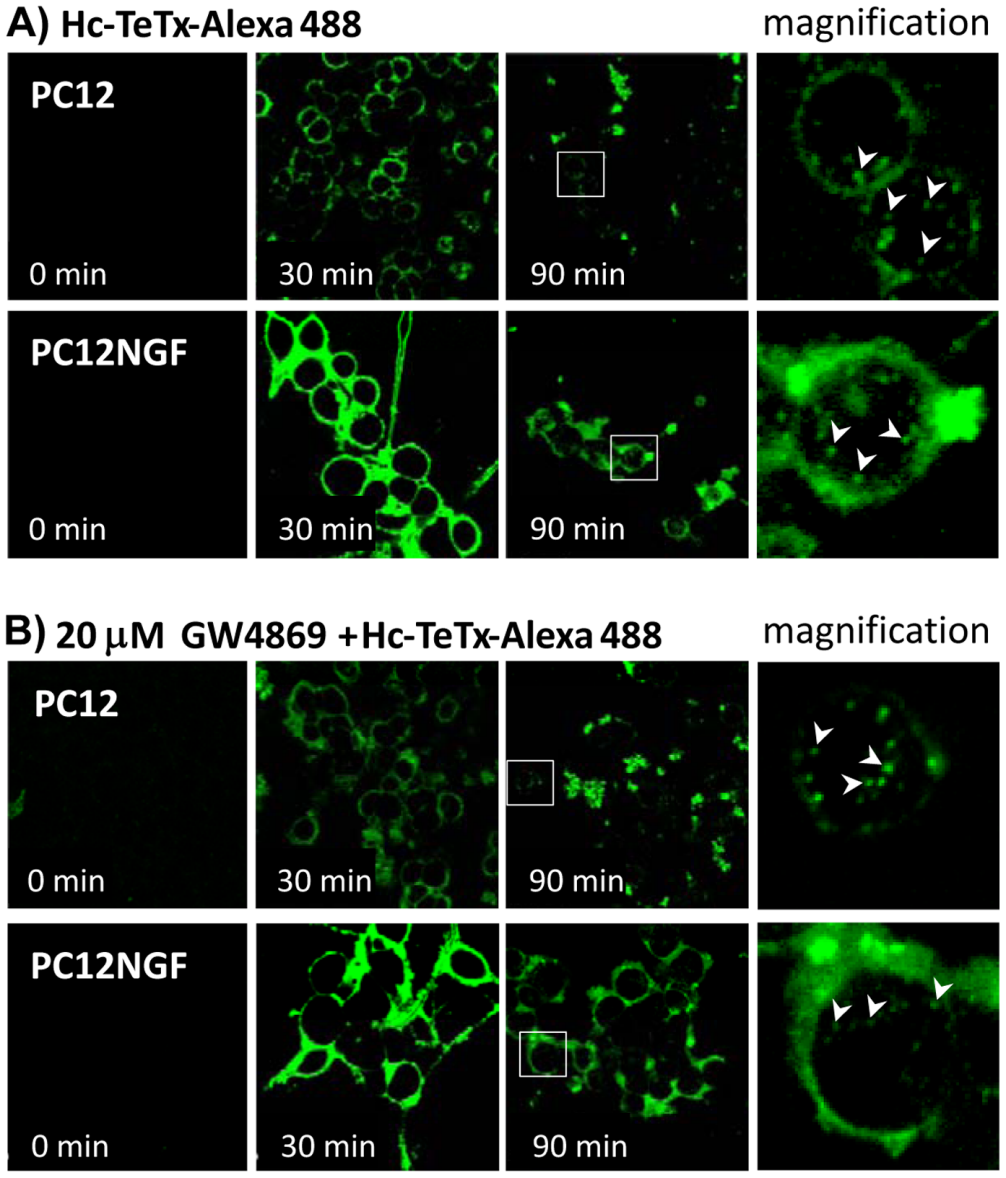
Neutral sphingomyelinase activity does not participate in Hc-TeTx internalization in PC12 cells. **A)** Undifferentiated (PC12) or NGF-differentiated PC12 (PC12NGF) cells were incubated Hc-Alexa 488 (1 nM) inside a thermostatic chamber at 37°C with CO_2_ supply and coupled to a confocal microscopy. The internalization was followed by time-lapse image capturing for 90 min. Hc-TeTx binds with much more avidity to PC12NGF than to PC12, as can be seen in the 30 min image, but internalization at this time point is not detectable in any case. At 90 min a similar degree of Hc internalization can be observed in both undifferentiated and differentiated cells. **B)** The same experiment was conducted with prior incubation of the cells with 20 µM GW4869 for 1 h. The results observed are not significantly different from the obtained in the absence of GW4869, indicating that nSMase activity and Hc internalization are independent events. Magnifications of the highlighted area are shown on the right, in all the cases, corresponding to the 90 min time point.

Since it has been described that some pathogens enhance sphingomyelinase activity [10], leading to the formation of ceramide platforms which are essential for the entry of the pathogen into the host cell [11], we decided to test whether the Hc-TeTx-enhanced nSMase activity and subsequent formation of ceramide platforms have a similar role. To do this, undifferentiated PC12 cells or NGF-differentiated PC12 cells were incubated with 1 nM Hc-TeTx-Alexa 488, in the absence or in the presence of GW4869. CGN cultures were discarded in these experiments because of its small cytoplasm, which greatly difficult the detection of internalized proteins. Hc-TeTx binding and internalization was followed in every case with time-lapse confocal microscopy for 1.5 hours. The images show that Hc-TeTx binds with much more avidity to NGF-differentiated PC12 cells than to non-differentiated cells, but the amount of internalized Hc seems to be similar in both cases, observing internalization at 90 min of incubation, but not at 30 min (Figure 8A). The pretreatment for 1 h with 20 µM GW4869 does not significantly affects the internalization of Hc-TeTx neither in undifferentiated PC12 nor in NGF-differentiated PC12 (Figure 8B), demonstrating the lack of nSMase implication in Hc-TeTx binding and internalization.

## Discussion

It is clear that pathogens can employ rafts, caveolae or raft-derived membrane domains to infect mammalian cells. Cases can be found in bacteriae, as *N. gonorrhoeae* [40], in viruses, as Rhinovirus (extensively reviewed in [41]), in parasites, as *P. falciparum* [42] and even in prionic diseases [43], among others. In some of these cases, the host membrane domains are not only used as binding platforms, but also are modified by enzymatic activities exerted or enhanced by the pathogen. A relevant example, seen in a series of cases, is the promotion of host sphingomyelinase activity, which causes an increase in the ceramide content in the plasmatic membrane. Several human rhinovirus strains induce the formation of functional ceramide membrane domains which are crucial components in the infection of host cells [11]. In the case of the measles virus, it has been demonstrated that the host immunesuppression caused in the host relies on the formation of ceramide platforms in T lymphocytes, impairing its cytoskeletal reorganization and function [44]. The mechanisms by which pathogens trigger ceramide production are not completely studied, but some interactions between pathogen membrane proteins and host receptors have been described. In the case of *N. gonorrhoeae*, a set of Opa proteins (surface components of this organism) has been shown to interact with members of the CEACAM (carcinoembryonic antigen cell adhesion molecule) receptor family on epithelial cells and on phagocytes. During *N. gonorrhoeae* infection, CEACAM-3 receptors are clustered in ceramide platforms, whose formation is enhanced by the pathogen itself, by means of host SMase activity promotion [40]. Moreover, Opa-mediated interactions lead to activation of the small GTPase Rac and of the stress-activated protein kinase JNK [45], which are critically involved in uptake of the bacteria. Internalization of *E. coli* is another event in which the pathogen induces clustering of host receptors in ceramide-enriched platforms, being in this case death receptors that lead to cell death [46]. In the case of *P. aeruginosa,* infection of epithelial cells results in a rapid translocation of aSMase onto the extracellular leaflet, mediating the formation of large ceramide-enriched platforms and internalization of the bacteria. Moreover, genetic deficiency of aSMase prevented the induction of apoptosis of infected cells and resulted in an uncontrolled release of cytokines, indicating that ceramide platforms might be involved in the negative regulation of signals initiated by pathogenic infections [8]. The results obtained in the studies on the biophysical behavior of ceramide-enriched membranes can provide the mechanistic basis of ceramide-dependent pathogen internalization. Regarding this, compiling evidences show that ceramide functions as a fusogen, being able to trigger the spontaneous fusion of ceramide-enriched microdomains into large ceramide-enriched macrodomains, since ceramide molecules have the tendency to interact with each other [47]. Studies in artificial lipid bilayers show that ceramide generated by enzymatic hydrolysis of SM in the upper leaflet results in bilayer asymmetry. This leads to lipid exchange between leaflets, to local enrichment of ceramides and to displacement of cholesterol towards outside ordered domains. This ceramide enrichment in the lower leaflet leads to a negative membrane curvature [48]. Furthermore, the SMase-driven generation of ceramide preserves the ability to fuse despite extensive cholesterol removal, probably by contributing to the total membrane negative curvature [49]. External addition of SMase using a micropipette in the vicinity of SM-containing giant unilamellar vesicles produced invaginations if SMase was externally added or budding if internally added [50]. Thus, promotion of host SMase activity by TeTx could be related with its necessary release to the intersynaptic space during the transynaptic jump that drives TeTx towards the inhibitory interneurons in the spinal cord. In any case, the promotion of ceramide-platforms by Hc-TeTx seems not to be essential for toxin internalization, as observed in the present work, but ceramide increase is related to Hc-enhanced signaling and prosurvival effects. Thus, apart from the ceramide role in membrane dynamics, its role in intracellular signaling is being an extensive field of research (for a review, see [51]). Studies on rhinoviruses internalization reveal that interactions between ceramide-enriched platforms and rhinoviruses are essential for signal transduction cascades employing phosphatidyl-inositol kinase, which activation is required for interleukin-8 expression and inflammation [52]. In previous work from our group, Hc-TeTx was described to prevent neuron loss under stress conditions, in *in vitro* cultures [32], [33] as well as in *in vivo* models [34], [35]. Thus, and taking into account that a moderate increase in ceramide levels leads to promotion of neuron survival [10], the observation of the increase ceramide by Hc-TeTx gains insight into part of the Hc-driven signaling mechanism used in the promotion of neuron resistance to stress. A mechanism leading to the same end is described for *Chlamydia,* which inhibits host cell apoptosis by accumulation of diacylglycerol in the inclusion vacuole and subsequent sequestering of PKCδ [53]. PKCδ is the member of the PKC family with the greatest number of described isoenzymes, generated by alternative splicing (from I to VIII), comprising either anti and proapoptotic isoforms [54]. In previous works from our group the induction of PKCδ translocation to membranes, an event related to its activation, was described by both TeTx [55] and Hc-TeTx [30], together with promotion of phospholipase Cγ-1 [30] and inositol phospholipid hydrolysis [56]. PKCδ has also been related to the formation of ceramide in the plasmatic membrane, since stimulated PKCδ is differentially trafficked to endolysosomes, causing phosphorylation and consequent targeting of acid SMase to the plasma membrane, where hydrolyzes SM to ceramide [57]. A prosurvival effect of ceramide is seen in the ischemic tolerance in cortical neurons due to sublethal oxygen-glucose deprivation [58]. This prosurvival effect of moderate increases in ceramide levels in cortical neurons is in agreement with the observation presented in figure 2C and with our previously published work [31]. Another clue pointing to the involvement of ceramide in prosurvival effects is the detection of increased amounts of C16, C24∶0 and C24∶1 ceramides in head and neck squamous cell carcinoma (HNSCC), respect to normal tissues, and associated with a higher incidence of lymphovascular invasion [59]. In any case, the ambivalent role of ceramides in cellular life and death has been emphasized thanks to the development of quantitative analytical methods to measure specific ceramides, becoming clear that the cellular effect of a ceramide molecule depends on its chain length (for a review see [60]). Thus, ceramide and its metabolic product, sphingosine, have been described as mainly antiproliferative and/or pro-apoptotic, a vision that would be in disagreement with the life-promoting activity of Hc. It is feasible, however, that ceramide represents an intermediate metabolite in the Akt-dependent cell viability promotion exerted by Hc, since ceramide and sphingosine can be converted to ceramide-1-phosphate (C1P) by ceramide kinase and to sphingosine-1-phosphate (S1P) by sphingosine kinase, respectively. C1P and S1P both promote growth and counteract apoptotic insults, by means of Akt activation in a PI3K-dependent manner [61].

Thus, the present work opens a new line of evidences on the TeTx actions in relation to two phenomena, i) the mechanism used by the toxin to interact with the target membrane and ii) the Hc-dependent signaling cascade that drives to host cell survival. Additionally, and no less important, is the demonstration that Hc increases the host cell viability under oxidative stress, a situation that is related to some pathologies. This last point greatly reinforces the role of the Hc fragment as a potential therapeutic tool to treat a number of pathological conditions, such as Parkinson’s disease, or, also, aging.

## References

[1] van der Meer-Janssen YPM, van Galen J, Batenburg JJ, Helms JB (2010) Lipids in host-pathogen interactions: Pathogens exploit the complexity of the host cell lipidome. Prog Lipid Res 49: 1–26. 19638285 10.1016/j.plipres.2009.07.003 PMC7112618

[2] Tsui-Pierchala BA, Encinas M, Milbrandt J, Johnson EM Jr (2002) Lipid rafts in neuronal signaling and function. TINS 25: 412–417. 12127758 10.1016/s0166-2236(02)02215-4

[3] Hanzal-Bayer MF, Hancock JF (2007) Lipid rafts and membrane traffic. FEBS Lett 581: 2098–2104. 17382322 10.1016/j.febslet.2007.03.019

[4] Pike LJ (2003) Lipid rafts: bringing order to chaos. J Lipid Res 44: 655–667. 12562849 10.1194/jlr.R200021-JLR200

[5] Riethmüller J, Riehle A, Grassmé H, Gulbins E (2006) Membrane rafts in host-pathogen interactions. Biochim Biophys Acta 1758: 2139–2147. 17094939 10.1016/j.bbamem.2006.07.017

[6] Abrami L, Liu S, Cosson P, Leppla SH, van der Goot FG (2003) Anthrax toxin triggers endocytosis of its receptor via a lipid raft-mediated clathrin-dependent process. J Cell Biol 160: 321–328. 12551953 10.1083/jcb.200211018 PMC2172673

[7] Herreros J, Ng T, Schiavo G (2001) Lipid rafts act as specialized domains for tetanus toxin binding and internalization into neurons. Mol Biol Cell 12: 2947–60. 11598183 10.1091/mbc.12.10.2947 PMC60147

[8] Grassmé H, Jendrossek V, Riehle A, von Kürthy G, Berger J, et al (2003) Host defense against Pseudomonas aeruginosa requires ceramide-rich membrane rafts. Nat Med 9: 322–30. 12563314 10.1038/nm823

[9] Grassmé H, Riehle A, Wilker B, Gulbins E (2005) Rhinoviruses infect human epithelial cells via ceramide-enriched membrane platforms. J Biol Chem 280: 26256–62. 15888438 10.1074/jbc.M500835200

[10] Posse de Chaves EI (2006) Sphingolipids in apoptosis, survival and regeneration in the nervous system. Biochim Biophys Acta 1758: 1995–2015. 17084809 10.1016/j.bbamem.2006.09.018

[11] Dreschers S, Franz P, Dumitru C, Wilker B, Jahnke K, et al (2007) Infections with human rhinovirus induce the formation of distinct functional membrane domains. Cell Physiol Biochem 20: 241–54. 17595532 10.1159/000104170

[12] Samet D, Barenholz Y (1999) Characterization of acidic and neutral sphingomyelinase activities in crude extracts of HL-60 cells. Chem Phys Lipids 102: 65–77. 11001562 10.1016/s0009-3084(99)00076-6

[13] Wu BX, Clarke CJ, Hannun YA (2010) Mammalian neutral sphingomyelinases: regulation and roles in cell signaling responses. Neuromolecular Med. 12: 320–30. 10.1007/s12017-010-8120-z PMC3405913 20552297

[14] Trajkovic K, Hsu C, Chiantia S, Rajendran L, Wenzel D, et al (2008) Ceramide triggers budding of exosome vesicles into multivesicular endosomes. Science 319: 1244–1247. 18309083 10.1126/science.1153124

[15] Halpern JL, Loftus A (1993) Characterization of the receptor-binding domain of tetanus toxin. J Biol Chem. 268: 11188–92. 8388386

[16] Lalli G, Bohnert S, Deinhardt K, Verastegui C, Schiavo G (2003) The journey of tetanus and botulinum neurotoxins in neurons. Trends Microbiol 11: 431–7. 13678859 10.1016/s0966-842x(03)00210-5

[17] Deinhardt K, Berninghausen O, Willison HJ, Hopkins CR, Schiavo G (2006) Tetanus toxin is internalized by a sequential clathrin-dependent mechanism initiated within lipid microdomains and independent of epsin. J Cell Biol 174: 459–471. 16880274 10.1083/jcb.200508170 PMC2064241

[18] Simpson LL (1980) Kinetic studies on the interaction between botulinum toxin type A and the cholinergic neuromuscular junction. J Pharmacol Expt Ther 212: 16–21. 6243359

[19] Dolly J O, Black J, Williams RS, Melling J (1984) Acceptors for botulinum neurotoxin reside on motor nerve terminals and mediate its internalization. Nature 307: 457–460. 6694738 10.1038/307457a0

[20] Montecucco C, Rossetto O, Schiavo G (2004) Presynaptic receptor arrays for clostridial neurotoxins. Trends Microbiol 12: 442–446. 15381192 10.1016/j.tim.2004.08.002

[21] Dong M, Yeh F, Tepp WH, Dean C Johnson EA, et al (2006) SV2 is the protein receptor for Botulinum Neurotoxin A. Science. 312: 592–596. 10.1126/science.1123654 16543415

[22] Peng L, Tepp WH, Johnson EA, Dong M (2011) Botulinum Neurotoxin D Uses Synaptic Vesicle Protein SV2 and Gangliosides as Receptors. PLoS Pathog 7(3): e1002008 doi:10.1371/journal.ppat.1002008 21483489 10.1371/journal.ppat.1002008 PMC3068998

[23] Dong M, Richards DA, Goodnough M, Tepp W, Johnson E, et al (2003) Synaptotagmins I and II mediate entry of botulinum neurotoxin B into cells. J Cell Biol 162: 1293–1303. 14504267 10.1083/jcb.200305098 PMC2173968

[24] Rummel A, Karnath T, Henke T, Bigalke H, Binz T (2004) Synaptotagmins I and II act as nerve cell receptors for botulinum neurotoxin G. J Biol Chem. 279: 30865–70. 10.1074/jbc.M403945200 15123599

[25] Yeh FL, Dong M, Yao J, Tepp WH, Lin G, et al (2010) SV2 Mediates Entry of Tetanus Neurotoxin into Central Neurons. PLoS Pathog 6(11): e1001207. 21124874 10.1371/journal.ppat.1001207 PMC2991259

[26] Munro P, Kojima H, Dupont JL, Bossu JL, Poulain B, et al (2001) High sensitivity of mouse neuronal cells to tetanus toxin requires a GPI-anchored protein. Biochem Biophys Res Commun 289: 623–629. 11716521 10.1006/bbrc.2001.6031

[27] Koriazova LK, Montal M (2003) Translocation of botulinum neurotoxin light chain protease through the heavy chain channel. Nat Struct Biol 10: 13–18. 12459720 10.1038/nsb879

[28] Schiavo G, Benfenati F, Poulain B, Rossetto O, Polverino de Laureto P, et al (1992) Tetanus and botulinum-B neurotoxins block neurotransmitter release by proteolytic cleavage of synaptobrevin. Nature 359: 832–835. 1331807 10.1038/359832a0

[29] Toivonen JM, Oliván S, Osta R (2010) Tetanus toxin C-fragment: the courier and the cure? Toxins 2: 2622–44. 22069568 10.3390/toxins2112622 PMC3153173

[30] Gil C, Chaïb-Oukadour I, Blasi J, Aguilera J (2001) Hc fragment (C-terminal portion of the heavy chain) of tetanus toxin activates protein kinase C isoforms and phosphoproteins involved in signal transduction. Biochem J 356: 97–103. 11336640 10.1042/0264-6021:3560097 PMC1221816

[31] Gil C, Chaib-oukadour I, Aguilera J (2003) C-terminal fragment of tetanus toxin heavy chain activates Akt and MEK/ERK signalling pathways in a Trk receptor-dependent manner in cultured cortical neurons. Biochem J 373: 613–620. 12710887 10.1042/BJ20030333 PMC1223507

[32] Chaïb-Oukadour I, Gil C, Aguilera J (2004) The C-terminal domain of the heavy chain of tetanus toxin rescues cerebellar granule neurones from apoptotic death: involvement of phosphatidylinositol 3-kinase and mitogen-activated protein kinase pathways. J Neurochem 90: 1227–1236. 15312177 10.1111/j.1471-4159.2004.02586.x

[33] Chaïb-Oukadour I, Gil C, Rodríguez-Alvarez J, Ortega A, Aguilera J (2009) Tetanus toxin Hc fragment reduces neuronal MPP^+^ toxicity. Mol Cell Neurosci 41: 297–303. 19344769 10.1016/j.mcn.2009.03.006

[34] Mendieta L, Venegas B, Moreno N, Patricio A, Martínez I, et al (2009) The carboxyl-terminal domain of the heavy chain of tetanus toxin prevents dopaminergic degeneration and improves motor behavior in rats with striatal MPP^+^-lesions. Neurosci Res 65: 98–106. 19523997 10.1016/j.neures.2009.06.001

[35] Moreno-Igoa M, Calvo AC, Penas C, Manzano R, Oliván S, et al (2010) Fragment C of tetanus toxin, more than a carrier. Novel perspectives in non-viral ALS gene therapy. J Mol Med 88: 297–308. 19921501 10.1007/s00109-009-0556-y

[36] Schutze K, Potthoff T, Machleidt D, Berkovic K, Wiegmann M, et al (1992) TNF activates NF-kappa B by phosphatidylcholine - specificphospholipase C - induced “acidic”. sphingomyelin breakdown, Cell 71: 765–776. 1330325 10.1016/0092-8674(92)90553-o

[37] Numakawa T, Nakayama H, Suzuki S, Kubo T, Nara F, et al (2003) Nerve Growth Factor-induced Glutamate Release Is via p75 Receptor, Ceramide, and Ca^2+^ from Ryanodine Receptor in Developing Cerebellar Neurons. J Biol Chem 278: 41259–41269. 12902347 10.1074/jbc.M304409200

[38] Albi E, Viola-Magni MP (2004) The role of intranuclear lipids. Biol Cell 96: 657–67. 15519699 10.1016/j.biolcel.2004.05.004

[39] Chai Y, Niu L, Sun XL, Ding JH, Hu G (2006) Iptakalim protects PC12 cell against H_2_O_2_-induced oxidative injury via opening mitochondrial ATP-sensitive potassium channel. Biochem Biophys Res Commun 350: 307–14. 17010314 10.1016/j.bbrc.2006.09.045

[40] Hauck CR, Grassmé H, Bock J, Jendrossek V, Ferlinz K, et al (2000) Acid sphingomyelinase is involved in CEACAM receptor-mediated phagocytosis of Neisseria gonorrhoeae. FEBS Lett 478: 260–266. 10930579 10.1016/s0014-5793(00)01851-2

[41] Takahashi T, Suzuki T (2011) Function of Membrane Rafts in Viral Lifecycles and Host Cellular Response. Biochem Res Int 2011, Article ID 245090, doi:10.1155/2011/245090. 10.1155/2011/245090 PMC3235436 22191032

[42] Murphy SC, Hiller NL, Harrison T, Lomasney JW, Mohanda N, et al (2006) Lipid rafts and malaria parasite infection of erythrocytes. Mol Memb Biol 23: 81–88. 10.1080/09687860500473440 16611583

[43] Baron GS, Wehrly K, Dorward DW, Chesebro B, Caughey B (2002) Conversion of raft associated prion protein to the protease- resistant state requires insertion of PrP-res [PrP(Sc)] into contiguous membranes. EMBO J 21: 1031–1040. 11867531 10.1093/emboj/21.5.1031 PMC125906

[44] Gassert E, Avota E, Harms H, Krohne G, Gulbins E, et al (2009) Induction of Membrane Ceramides: A Novel Strategy to Interfere with T Lymphocyte Cytoskeletal Reorganisation in Viral Immunosuppression. PLoS Pathog 5: e1000623. 19834551 10.1371/journal.ppat.1000623 PMC2757718

[45] Hauck CR, Meyer TF, Lang F, Gulbins E (1998) CD66-mediated phagocytosis of Opa52 *Neisseria gonorrhoeae* requires a Src-like tyrosine kinase- and Rac1-dependent signaling pathway. EMBO J 17: 443–454. 9430636 10.1093/emboj/17.2.443 PMC1170395

[46] Falcone S, Perrotta C, De Palma C, Pisconti A, Sciorati C, et al (2004) Activation of acid sphingomyelinase and its inhibition by the nitric oxide/cyclic guanosine 3′,5′-monophosphate pathway: key events in Escherichia coli-elicited apoptosis of dendritic cells. J Immunol 173: 4452–63. 15383576 10.4049/jimmunol.173.7.4452

[47] Veiga MP, Arrondo J, Goñi FM, Alonso A (1999) Ceramides in phospholipid membranes: effects on bilayer stability and transition to nonlamellar phases. Biophys J 76: 342–350. 9876146 10.1016/S0006-3495(99)77201-2 PMC1302523

[48] Ira, Johnston LJ (2008) Sphingomyelinase generation of ceramide promotes clustering of nanoscale domains in supported bilayer membranes. Biochim Biophys Acta 1778: 185–197. 17988649 10.1016/j.bbamem.2007.09.021

[49] Rogasevskaia T, Coorssen JR (2006) Sphingomyelin-enriched microdomains define the efficiency of native Ca^2+^-triggered membrane fusion. J Cell Sci. 119: 2688–2694. 10.1242/jcs.03007 16757517

[50] Holopainen JM, Angelova MI, Kinnunen PKJ (2000) Vectorial budding of vesicles by asymmetrical enzymatic formation of ceramide in giant liposomes. Biophys J 78: 830–838. 10653795 10.1016/S0006-3495(00)76640-9 PMC1300685

[51] Ruvolo PP (2003) Intracellular signal transduction pathways activated by ceramide and its metabolites. Pharm Res 47: 383–392. 10.1016/s1043-6618(03)00050-1 12676512

[52] Bentley JK, Newcomb DC, Goldsmith AM, Jia Y, Sajjan US, et al (2007) Rhinovirus activates IL-8 expression via a Src/p110β PI 3-kinase/Akt pathway in human airway epithelial cells. J Virol 81: 1186–94. 17121804 10.1128/JVI.02309-06 PMC1797503

[53] Tse SML, Mason D, Botelho RJ, Chiu B, Reyland M, et al (2005) Accumulation of Diacylglycerol in the Chlamydia Inclusion Vacuole. J Biol Chem 280: 25210–25215. 15863503 10.1074/jbc.M501980200

[54] Jiang K, Apostolatos A, Ghansah T, Watson JE, Vickers T, et al (2008) Identification of a Novel Antiapoptotic Human Protein Kinase C δ Isoform, PKCδVIII in NT2 Cells. Biochemistry 47: 787–797. 18092819 10.1021/bi7019782

[55] Gil C, Chaïb-Oukadour I, Pelliccioni P, Aguilera J (2000) Activation of signal transduction pathways involving trkA, PLCγ-1, PKC isoforms and ERK-1/2 by tetanus toxin. FEBS Lett 481: 177–182. 10996319 10.1016/s0014-5793(00)02002-0

[56] Gil C, Ruiz-Meana M, Alava M, Yavin E, Aguilera J (1998) Tetanus Toxin Enhances Protein Kinase C Activity Translocation and Increases Polyphosphoinositide Hydrolysis in Rat Cerebral Cortex Preparations. J Neurochem 70: 1636–1643. 9523581 10.1046/j.1471-4159.1998.70041636.x

[57] Zeidan YH, Hannun YA (2007) Activation of Acid Sphingomyelinase by Protein Kinase Cδ-mediated Phosphorylation. J Biol Chem 282: 11549–11561. 17303575 10.1074/jbc.M609424200

[58] Bhuiyan M, Islam M, Jung S, Yoo H, Yee Y, et al (2010) Involvement of Ceramide in Ischemic Tolerance Induced by Preconditioning with Sublethal Oxygen-Glucose Deprivation in Primary Cultured Cortical Neurons of Rats. Biol Pharm Bull 33: 11–17. 20045928 10.1248/bpb.33.11

[59] Karahatay S, Thomas K, Koybasi S, Senkal CE, Elojeimy S, et al (2007) Clinical relevance of ceramide metabolism in the pathogenesis of human head and neck squamous cell carcinoma (HNSCC): attenuation of C(18)-ceramide in HNSCC tumors correlates with lymphovascular invasion and nodal metastasis. Cancer Lett 256: 101–11. 17619081 10.1016/j.canlet.2007.06.003 PMC2084356

[60] Grösch S, Schiffmann S, Geisslinger G (2012) Chain length-specific properties of ceramides. Prog Lipid Res 51: 50–62. 22133871 10.1016/j.plipres.2011.11.001

[61] Gómez-Muñoz A, Kong JY, Parhar K, Wang SW, Gangoiti P, et al (2005) Ceramide-1-phosphate promotes cell survival through activation of the phosphatidylinositol 3-kinase/protein kinase B pathway. FEBS Lett 579: 3744–50. 15978590 10.1016/j.febslet.2005.05.067

